# A high-content AlphaScreen™ identifies E6-specific small molecule inhibitors as potential therapeutics for HPV^+^ head and neck squamous cell carcinomas

**DOI:** 10.18632/oncotarget.27908

**Published:** 2021-03-16

**Authors:** Lennox Chitsike, Chung-Hsiang Yuan, Anuradha Roy, Kristopher Boyle, Penelope J. Duerksen-Hughes

**Affiliations:** ^1^Department of Basic Science, School of Medicine, Loma Linda University, Loma Linda, CA, USA; ^2^High-Throughput Screening Laboratory, University of Kansas, Lawrence, KS, USA; ^3^School of Pharmacy, Loma Linda University, Loma Linda, CA, USA

**Keywords:** HNSCC, HPV, E6, AlphaScreen™, 30-hydroxygambogic acid

## Abstract

The incidence of human papillomavirus-positive head and neck squamous cell carcinoma (HPV^+^-HNSCC) has increased dramatically over the past decades due to an increase in infection of the oral mucosa by HPV. The etiology of HPV^+^-HNSCC is linked to expression of the HPV oncoprotein, E6, which influences tumor formation, growth and survival. E6 effects this oncogenic phenotype in part through inhibitory protein-protein interactions (PPIs) and accelerated degradation of proteins with tumor suppressor properties, such as p53 and caspase 8. Interfering with the binding between E6 and its cellular partners may therefore represent a reasonable pharmacological intervention in HPV^+^ tumors. In this study, we probed a small-molecule library using AlphaScreen™ technology to discover novel E6 inhibitors. Following a cascade of screens we identified and prioritized one hit compound. Structure activity relationship (SAR) studies of this lead uncovered an analog, 30-hydroxygambogic acid (GA-OH), that displayed improved activity. Further testing of this analog in a panel of HPV^+^ and HPV^–^ cell lines showed good potency and a large window of selectivity as demonstrated by apoptosis induction and significant inhibition of cell growth, cell survival in HPV^+^ cells. In summary, GA-OH may serve as a starting point for the development of potent E6-specific inhibitors.

## INTRODUCTION

Head and neck squamous cell carcinomas (HNSCC) are heterogeneous tumors that arise in the upper respiratory tract and are the 6th most common cancer worldwide by incidence. The two main subtypes of HNSCC, HPV^-^-HNSCC and HPV^+^-HNSCC, are distinct and diverging in their features. HPV^-^-HNSCC, historically caused by chemical carcinogens such as alcohol and tobacco, has been in decline for the past 3 decades. In parallel, HPV^+^-HNSCC, which is caused by HPV, has risen dramatically (over 225%) within the same period [1–3]. Changes in sexual practices, particularly in Western countries, has increased the colonization of the oral mucosa by HPV, along with the associated malignancies. The advent of HPV vaccines has the potential to prevent this number from climbing upwards in the next decades. However, even with the availability of vaccines, the burden of HPV^+^-HNSCC will remain a concern in the future due to limited uptake of the vaccines. In addition, the vaccine is ineffective in those already infected [4–6]. For patients already presenting with HNSCC, current treatment guidelines recommend a combination approach involving surgery, radiation and chemotherapy, irrespective of the HPV status [7, 8]. This approach is not optimal, given the known distinct tumor biology and response to therapy. Compared to its HPV^-^ counterpart, HPV^+^-HNSCC carries a more favorable prognosis and is more prevalent in younger and otherwise healthier patients [7, 8]. Importantly, the use of such standard therapies is associated with debilitating life-long morbidities [1, 9]. Taken together, there has been a general consensus that the HPV^+^ subgroup may be over-treated and that selective therapies that spare patients from these long-term and deleterious side effects are needed.

Innovative and safer therapeutic strategies such as targeted therapies are needed to safely combat the growing HPV^+^-HNSCC epidemic. HPV oncoproteins, particularly E6, represent a unique and potentially therapeutically favorable strategic approach for targeted HPV^+^-HNSCC treatment. E6 is a causative agent in the cellular transformation and immortalization of keratinocytes, and its continuous expression is necessary to maintain tumor progression [9]. E6 also modulates the survival of HPV^+^ tumor cells by impacting how they respond to apoptotic stimuli. This occurs primarily through inhibitory protein-protein interactions with proteins such as p53 and caspase 8. [7, 10–12] Studies in our lab have previously demonstrated that E6 directly binds to proteins in the extrinsic apoptotic pathway such as caspase 8 [13–15]. Similar observations, showing E6 physically binding to proteins of the intrinsic apoptosis pathway such as p53 and Bak, and consequently facilitating their proteasomal degradation, have also been reported [16, 17]. Furthermore, we have studied the therapeutic implications of this protein-protein binding and demonstrated that such E6-mediated inhibition of caspase 8 blunts the induction of cell death of HPV^+^ cells by apoptosis-inducing cancer therapies [18]. These findings are corroborated by findings that have shown that the absence of p53 and caspase 8 in HNSCC is correlated with attenuation of sensitivity of HPV^+^-HNSCC to chemotherapy and radiation [19–21]. Consistent with this, genetic tools such as CRISPER, TALEN gene knockouts, RNAi and other agents that indirectly knock down E6 mRNA have demonstrated that depleting the protein abundance of E6 leads to anti-proliferative effects and enhances the response of HPV^+^ cells to chemotherapy agents and radiation [11, 12]. Collectively, these studies show that E6 acts by blocking apoptosis, and that its critical role as a survival factor in HPV^+^ tumors make it an attractive therapeutic target.

Herein we describe our search for small molecule inhibitors that disrupt binding of E6 to caspase 8 using AlphaScreen technology™ (Perkin Elmer, Waltham, MA). This technology is a proximity-based platform for identifying hit compounds that perturb a specific interaction between two beaded proteins. Using this approach, we interrogated a library of over 5000 small molecules for compounds that antagonize E6 binding to caspase 8. We identified 96 hits, then further characterized them through a number of complementary and orthogonal tests to authenticate their activity and specificity. GA-OH emerged as the most promising inhibitor of E6, and in follow-up cell-based studies, showed selective growth suppression and increased cell death. These results suggest strategies for the development of novel therapies for HPV^+^-HNSCC.

## RESULTS

### Primary screening and hit identification

**Figure 1:**
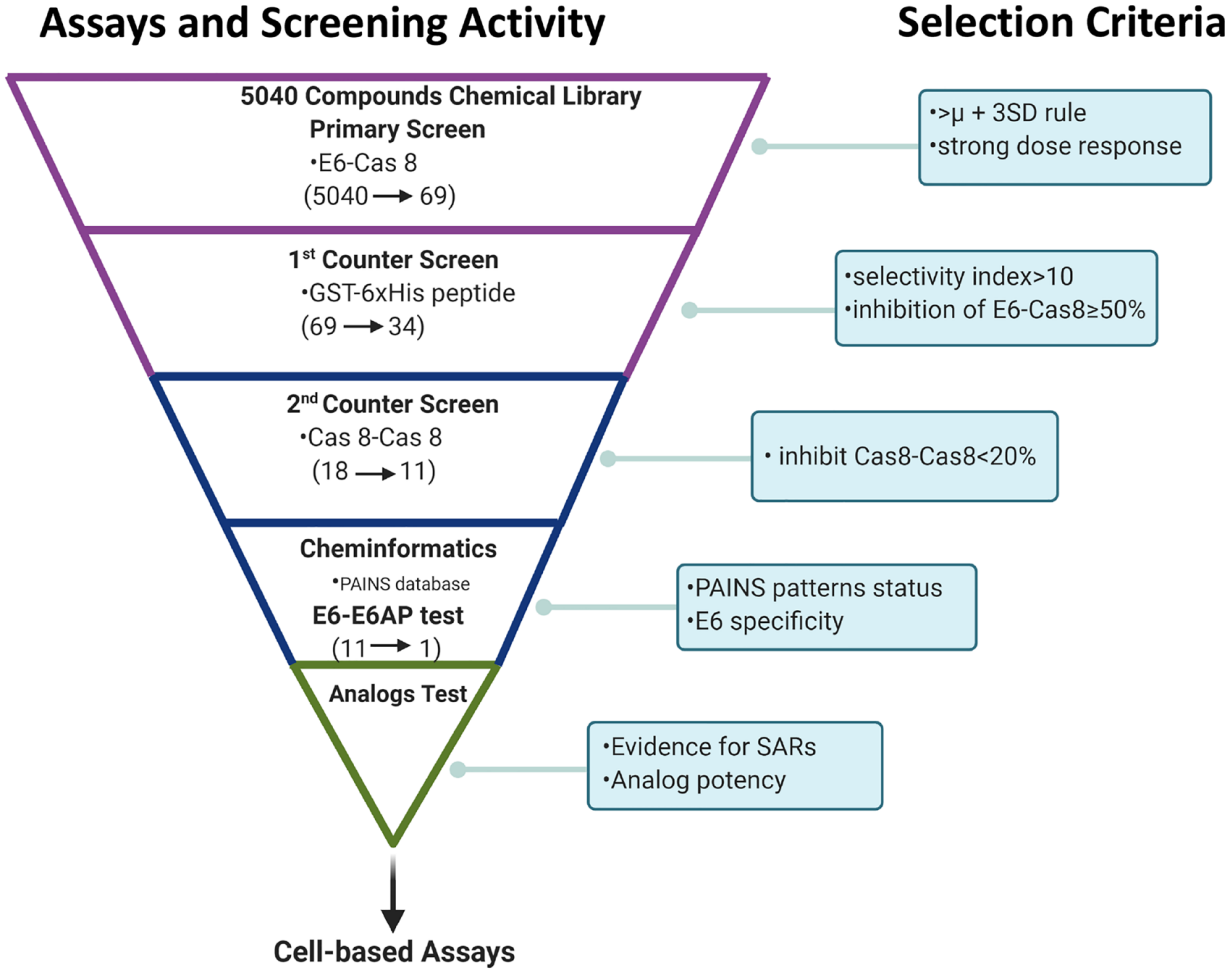
Summary of high content screening strategy. Screening funnel scheme shows the screening activity performed at each given step of hit compound triage and the respective decision criterion used for hit selection. Primary screening was followed by secondary screening and cheminformatics filtering using PAINS databases. Relationships between structure and activity were then analyzed before one candidate was chosen for cell studies. (Scheme created with https://www.BioRender.com).

**Figure 2:**
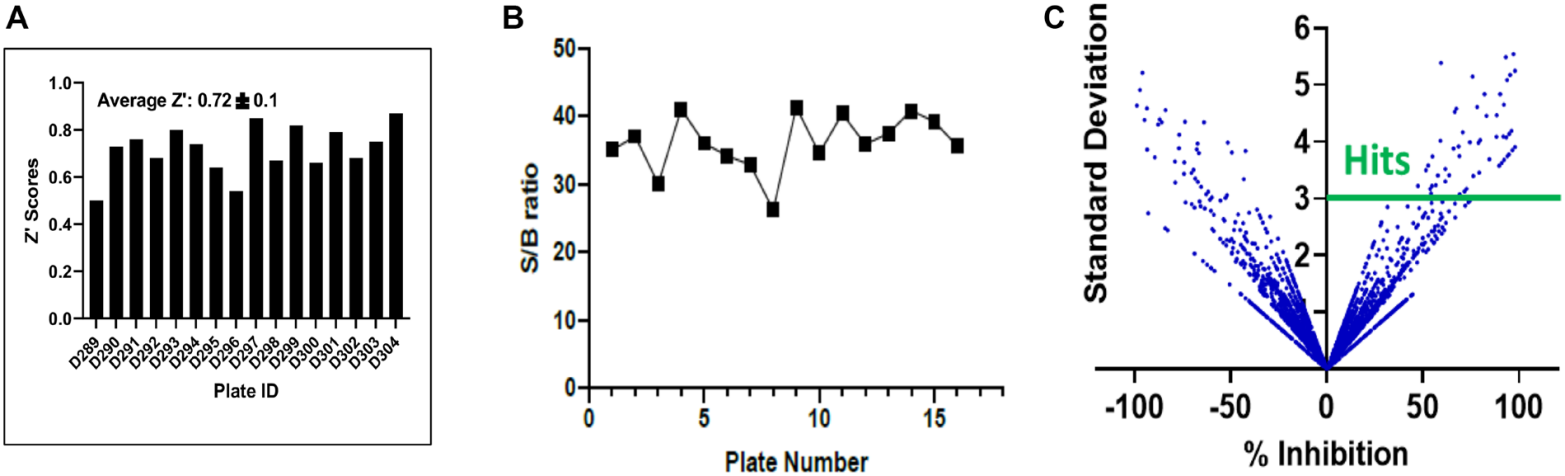
Statistical validation of the screening assay and hit selection. (**A**) Z factor scores were determined for each plate, and the median and average scores were found to both be > 0.6, indicating suitability of the assay for high content screening. (**B**) The assay demonstrated excellent sensitivity as shown by the high signal to background ratio for each plate. (**C**) Hits were selected using a computed cut off value of % Inhibition 3SD rule, as shown in this scatter plot compound (compounds above the solid green line).

We screened 3 structurally diverse libraries (Prestwick library, Microsource Spectrum library and an in-house collection at Kansas University; see Supplementary Table 1) for the ability of compounds to inhibit the binding of full-length HPV E6 to human Caspase 8 using a previously optimized AlphaScreen™ protocol [22]. Our overall hit selection workflow is summarized in Figure 1. For each of the compounds screened, % inhibition was calculated. A histogram plot of all the compounds against their % inhibition displayed a normal distribution. We next calculated the following parameters: Z-factor, S/B ratio and % CV to evaluate the assay quality and performance. The resulting statistical parameters from our screening data indicated good statistical validation and adequate suitability of the assay for high content screening (Figure 2). Z’-factor > 0.5 are considered the threshold for the assay to be considered excellent and suitable for high content screening. The median and mean Z’ factors that we calculated for the 16 384-well plates that were used to screen our 5k library were 0.72 and 0.67, respectively. These Z’ factor scores demonstrate suitability for high throughput screening. Similarly, the assay also demonstrated high sensitivity with the mean S/B ratio of 36 (Figure 2B). Variability between plates was also low for all the 16 plates with the mean CV of 9.6%, and well below the acceptable threshold of < 20%. With our quality control parameters well within the acceptable ranges and suggesting overall robustness, we focused on identifying possible hit compounds. Standard deviation from sample mean for each compound was plotted against % inhibition of E6 binding to caspase 8. We applied the μ + 3SD rule to our normalized % inhibition data, and compounds that were 3 Z-scores above the sample average were selected (Figure 2C). With this hit selection cutoff, 96 compounds were selected as preliminary hits for an initial hit rate of about 1.9%. These 96 compounds were then subjected to dose-response analysis to assess competitive behavior as well as the relationship between concentration and inhibitory activity on E6 binding. Of the initial hits, 69 displayed a strong dose response as demonstrated by clear sigmoidal behavior and IC_50_ values around 10 μM or lower (Supplementary Table 2), and were thus selected for secondary screening as discussed below.

### Counter-screening and hit confirmation

**Figure 3:**
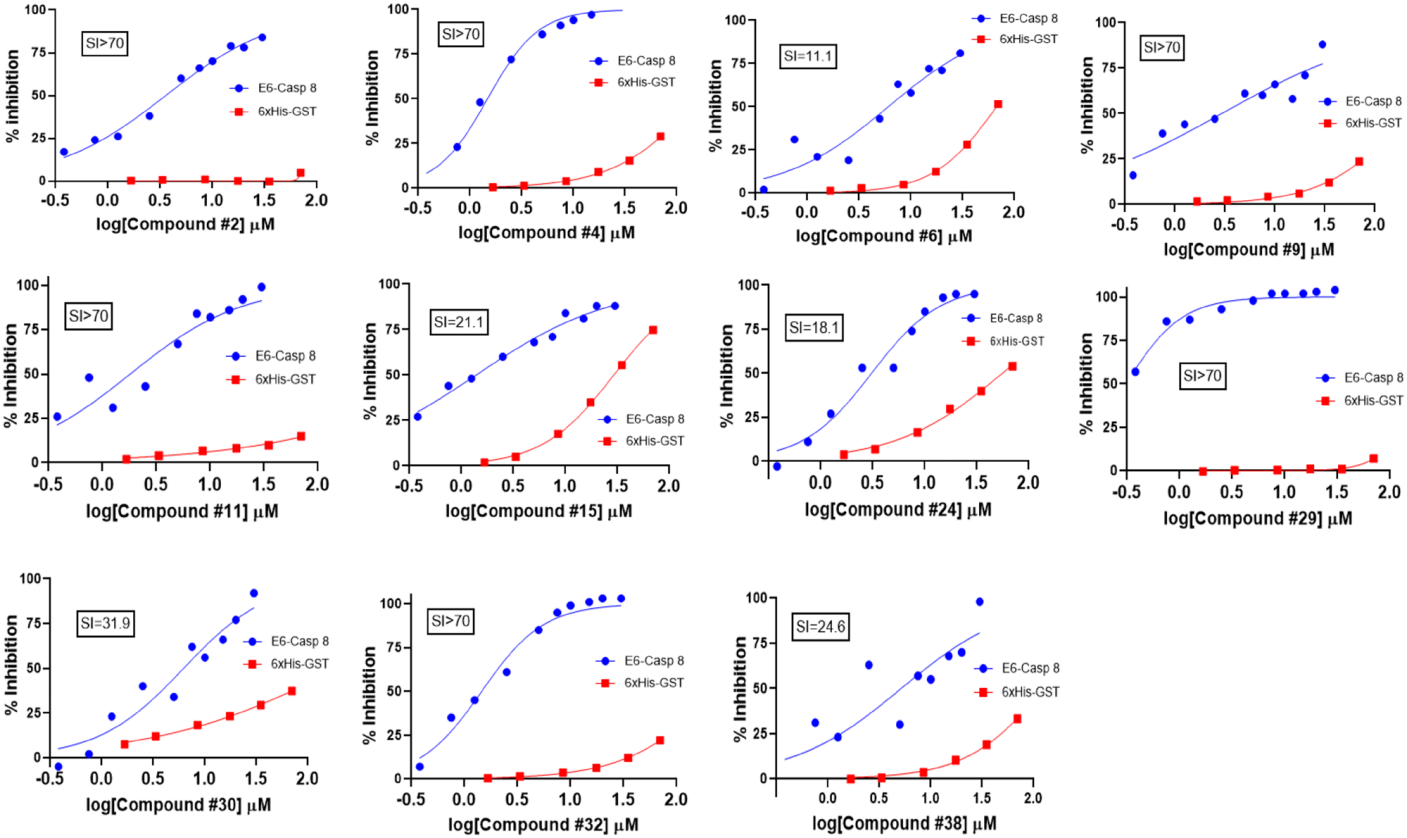
Counter-screening of initial hits. Binding activity graphs for the 11 compounds that passed both the 6xHis-GST and Caspase 8-Caspase 8 counter-screens are shown. The graphs show activity of each compound against Caspase 8-E6 and 6xHis-GST binding, and the calculated SI values from the activity against these two types of substrates (IC_50_/IC_50_) are shown.

In AlphaScreen™, artifacts that interfere with aspects of signal generation and bead capture, rather than the binding of the two proteins being assayed, may initially identify as hits. A counter-screen is necessary to eliminate compounds with such non-specific and promiscuous interactions. To do this, we employed two distinct counter-screens. For the first, we utilized the GST-6xHis fusion peptide. This peptide, comprising the affinity handles of E6 and caspase 8 respectively, represented the null control reaction. To assess specificity, we also ran the primary screen (E6-Caspase 8) in parallel. From the null and primary reactions we calculated the selectivity index (SI), and compounds with preferential inhibition of E6-Caspase 8 relative to GST-His_6_ were chosen; the rest were removed from consideration as promiscuous. Thirty-four of the initial hit compounds displayed an SI > 10; that is, about 50% of the initial hits were at least 10-fold more selective in inhibiting E6-Caspase 8 binding versus the control substrate. Conversely, about half of the compounds were identified as frequent hitters and thus non-selective. From these remaining hits, 18 compounds were cherry-picked based on commercial availability and strength of selectivity index, as well as whether their maximum inhibition of E6-Caspase binding was ≥ 50%. We then subjected these compounds to the second counter-screen. This counter-screen assessed the ability of compounds to interfere with GST-Caspase 8-His_6_-Caspase 8 binding, rather than GST-E6-His_6_-Caspase 8 binding, and its objective was to flag compounds that preferentially bind to caspase 8 rather than to E6, potentially interfering with host cell apoptosis. The inclusion criteria for the preferred compounds in this screen was set to less than 20% inhibition of Caspase 8-Caspase 8 binding. Using this criterion, 11 of the 18 compounds were taken as “true” primary hits for a confirmed hit rate of 0.22%. The selectivity profiles and indices of the 11 compounds are shown in Figure 3. The IC_50_ values of these compounds against E6-caspase 8 binding are also shown in Supplementary Table 3. The binding profiles show that these compounds exhibit little to no interference with the assay itself. In addition, these compounds also show little interaction with the dimerization of caspase 8 as shown in Supplementary Figure 1. These results indicate that they are specific inhibitors of the interaction between E6 and caspase 8.

### Cheminformatic analysis

**Figure 4:**
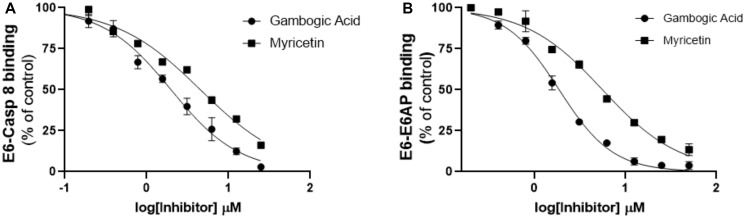
Follow-up characterization of specificity and activity of gambogic acid for E6 binding inhibition. Head-to-head comparison of the lead compound (gambogic acid) with myricetin against binding of E6 to (**A**) Caspase 8 and (**B**) E6AP. The inhibitory activity of gambogic acid as measured by AlphascreenTM is superior to that of myricetin, which we have previously shown to inhibit E6 binding.

To prioritize the remaining 11 hit compounds for downstream analysis such as SAR and cell-based functional studies, we took a more qualitative cheminformatic approach. Our goal was to prioritize compounds with no known promiscuity in biochemical assays by looking for the presence of PAINS patterns. Using four PAINS-detector online tools that recognize substructures of frequent hitter compounds, we excluded any flagged compound and selected those that came out as PAINS-free in all four runs. This analysis was complemented by a qualitative examination of the remaining compounds to flag bad functional groups (BFGs) or problematic substructures that could have been missed computationally. Comparison with the literature enabled us to identify and pursue only those compounds that possessed novel and unreported activity against E6 [22–26]. After these steps, gambogic acid (compound #24) remained the best candidate for further studies. We then cross-validated its activity by performing additional AlphaScreening™ using GST-E6 and His_6_-E6AP as substrates. Our previous published findings had shown that myricetin, another E6 inhibitor, prevents binding of both caspase 8 and E6AP to E6 [22, 24]. We therefore included myricetin as a positive control in this assay. We also evaluated the activity of these two inhibitors against E6-Caspase 8 binding in parallel for a head to head comparison. Compared to myricetin, gambogic acid displayed greater potency than myricetin against binding to both substrates with inhibitory concentrations that were at least two-fold lower (IC_50_ 1.9 μM vs. 4.6 μM against E6-Caspase 8 and IC_50_ 1.7 μM vs. 5.6 μM against E6-E6AP). These findings suggest potential for rescuing caspase 8 and p53 functions in cells (Figure 4A and 4B) [22, 24].

### SAR analysis

**Figure 5:**
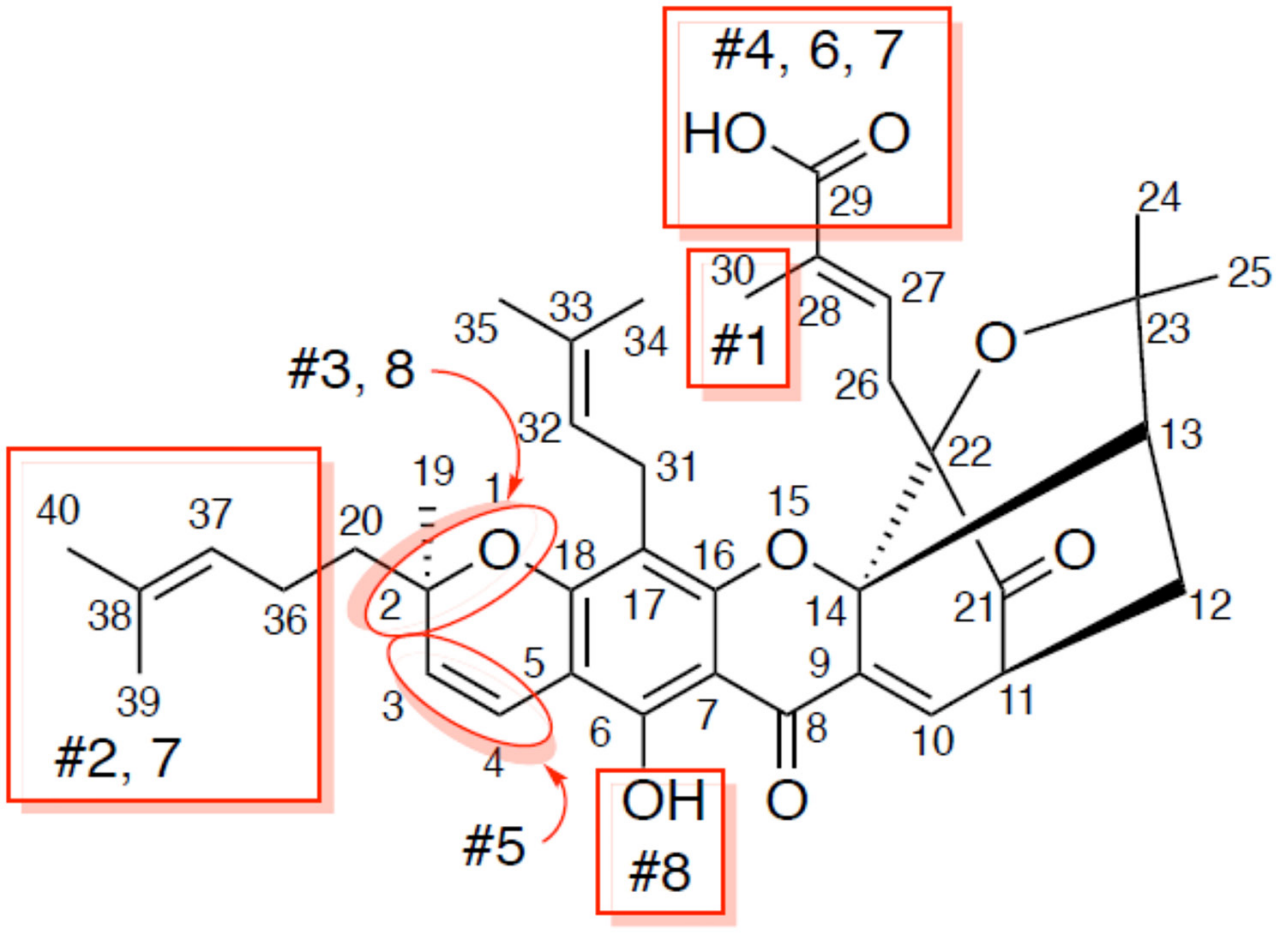
Analogs of gambogic acid (structure-activity relationship study). Rings A, B, C, D make up the core scaffold of gambogic acid. Analog #1 is 30 hydroxy gambogic acid (GA-OH), analog #2 is morellic acid, analog #3 is gambogenic acid, analog #4 is gambogic amide, analog #5 is neo-gambogic acid, analog #6 is gambogin, analog #7 is iso morellinol, and analog #8 is acetyl iso-gambogic acid.

**Figure 6:**
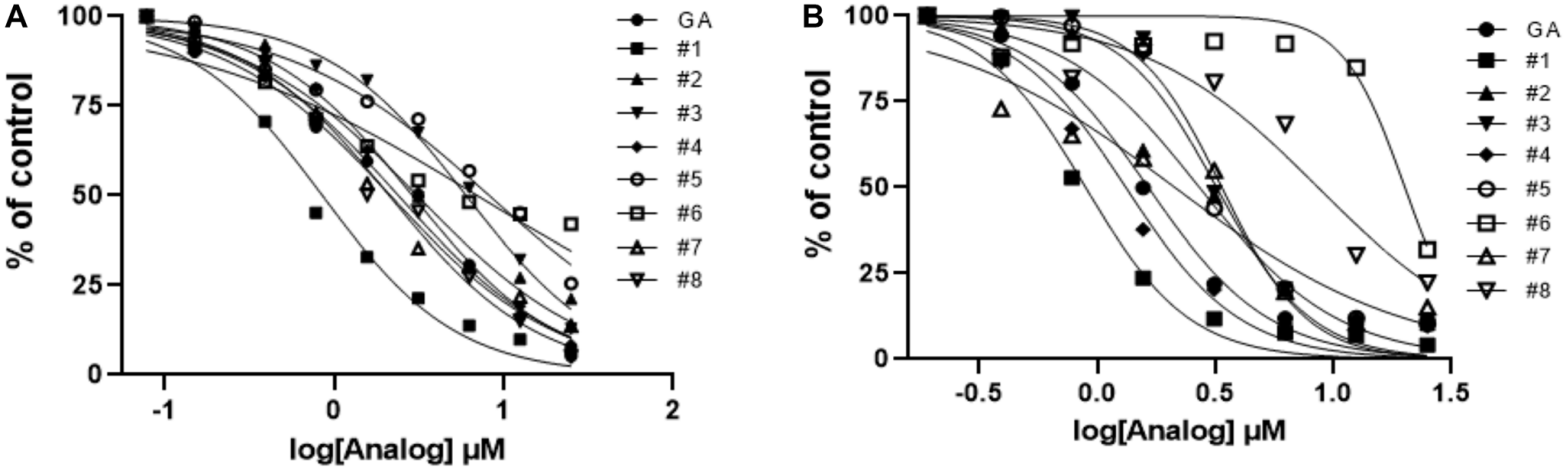
Activity profiles of analogs of gambogic acid *in vitro* (AlphaScreen) and *in vivo* (HPV^+^ cell line assays). (**A**) Evaluation of E6 hit analog activity *in vitro* using E6-Caspase 8 substrates. With the exception of # 3, 5, and 6, most analogs show activity close to that of the parent compound. (**B**) In the *in vivo* (cells) context, #3, 5, and 6 also show low activity; #8 is a surprise addition to this group. #1 remains the most promising lead in terms of activity in the HPV^+^ cell line.

Guided by this information, we next purchased 8 analogs of gambogic acid from commercial vendors to carry out a limited structure-activity relationship (SAR) analysis (Figures 5 and 6). We performed AlphaScreen™ analysis of these structural analogs for their ability to inhibit E6-caspase 8 binding. Generally, the differences in inhibitory activity amongst the analogs were not dramatic, which is not surprising due to their structural similarity (Figures 5 and 6). That said, the activity of three of these analogs (#3, 5, and 6) deviated noticeably (~ >3 logs less active) from the parent compound. Analogs #3 and 5 had modifications to ring A from the core scaffold of gambogic acid. Analog #3 had the A ring cleaved, while analog #5 had the C3/4 olefin oxidized and a hydroxyl group at C4. The modification on analog #6 was distant from the core ring system, with the compound lacking the carboxylic acid at C29, possessing instead an unoxidized methyl group. The structural changes represented by analogs #2 (removal of the isoprenyl group at C2), #4 (replacement of the carboxylic acid at C29 with a primary amide), #7 (removal of the isoprenyl at C2 and reduction of the C29 carboxylic acid to an alcohol) and #8 (acetylation of the C8 phenol and epimerization at C2) did not significantly affect the activity of the analogs relative to GA. Notably, analog #1, 30-hydroxygambogic acid, more effectively inhibited E6 binding to caspase 8 than any of the other analogs or the parent compound. The increased binding of analog #1, possessing a hydrogen-bonding hydroxyl group at C30, and the loss of activity of analog #6, which removed the carboxylic acid at C29, indicate the importance of the oxidized isoprenyl group at C22 to the activity of these compounds against E6. The unaffected activity of C29 amide analog #4 is also significant.

Next, we tested the analogs for functional activity using the HPV^+^ HNSCC cell line, SCC104, using the MTT assay as described previously to determine whether similar findings would be observed as in the *in vitro* AlphaScreen™ analysis. With one exception, the patterns we observed with in our AlphaScreen™ data were maintained, but the differences were relatively more pronounced in the cell-based screen (Figure 6B). Analogs #3, 5 and 6 had the third, fourth and first highest IC_50s_ relative to the parent compound, respectively. The only compound that significantly differs from the trend seen in the AlphaScreen™ results is analog #8, which showed high activity *in vitro* but diminished activity in this cell-based assay. As in the AlphaScreen™ analysis, analog #1 (30-hydroxygambogic acid) showed the highest potency of all analogs, including the parent compound. Based on these findings, we selected 30-hydroxygambogic acid (GA-OH) as our candidate for more extensive functional studies.

### GA-OH selectively inhibits cell growth and cell survival in HPV^+^ cell lines

**Figure 7:**
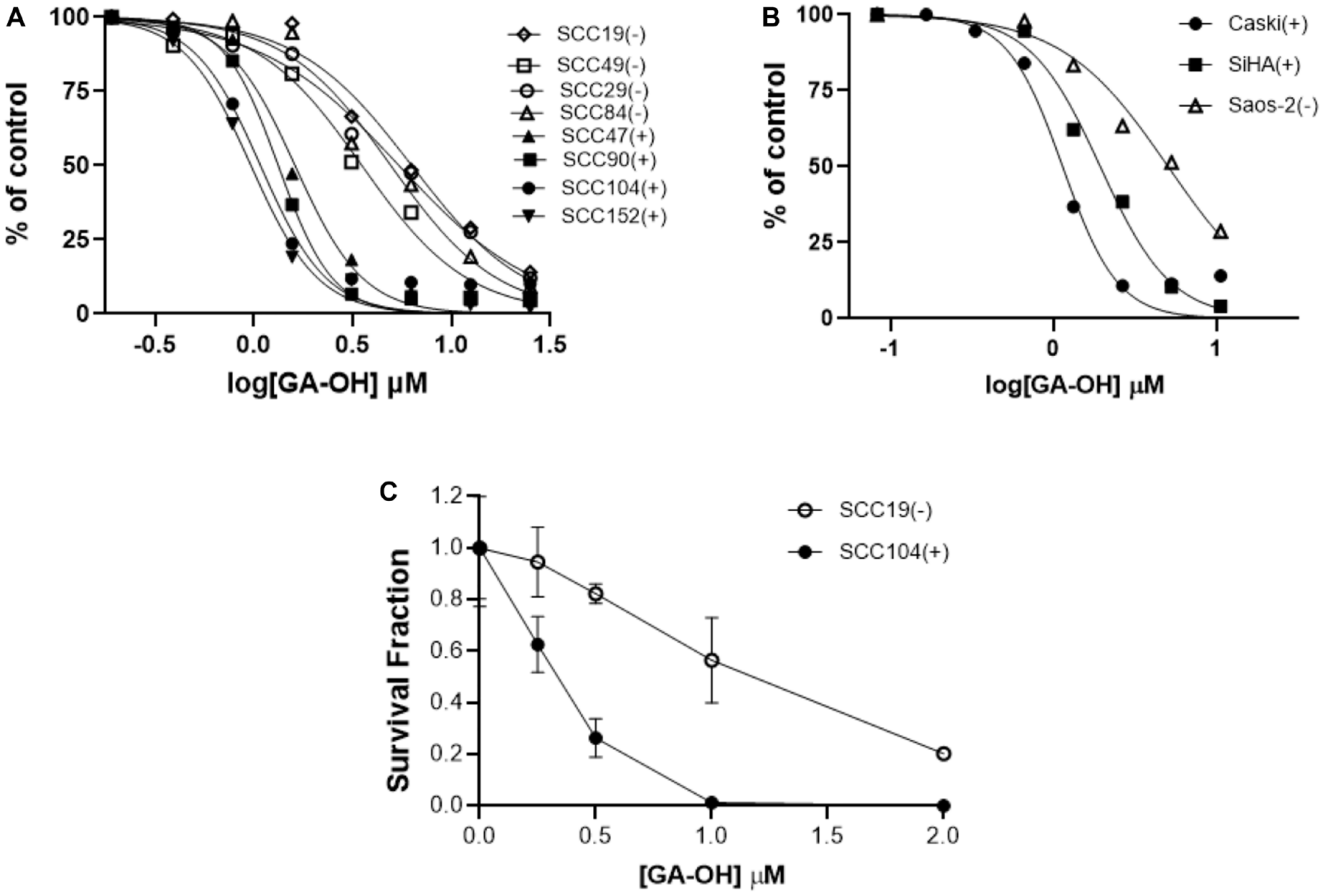
HPV^+^ cell lines display higher sensitivity to GA-OH-mediated growth inhibition than do HPV- cell lines. (**A**, **B**) Cell growth inhibition of HNSCC (A) and cervical cancer (B) HPV^+^ and HPV^-^ cell lines by GA-OH. HPV^+^ cell lines display higher sensitivity than do HPV^-^ cell lines as shown by the leftwards shift of the HPV^+^ curves (SCC47, 90, 104, 152 in A) and SiHa and CaSki in B). (**C**) Effects of GA-OH on the clonogenicity of the surviving fractions of HPV^+^ versus HPV^-^ cell lines. GA-OH displayed higher cytotoxicity to HPV^+^ cells. (+ and closed shapes represent HPV^+^ cell lines and – and open shapes the HPV^-^ cell lines).

Although SAR studies using the SCC104 cell line gave evidence of the activity of the analogs, it was critical to assess not just the potency, but also the selectivity. As a step towards that goal, we evaluated the efficacy of GA-OH in a panel containing both HPV^+^ and HPV^-^ HNSCC cell lines using MTT cell viability assays. Four HPV^+^ cell lines (SCC47, SCC090, SCC104, SCC152) and four HPV^-^ cell lines (SCC19, SCC29, SCC49, SCC84) were utilized. GA-OH behaves dose dependently in cell lines both with or without HPV. However, the HPV^+^ cell lines tested here displayed higher sensitivity than did the HPV^-^ cell lines (Figure 7A). These differentials in activity between HPV^+^ and HPV^-^ cell lines were consistent with our working model, given our AlphaScreen™ results that showed evidence that GA-OH could inhibit E6 interactions with pro-apoptotic molecules such as caspase 8 and E6AP. To further validate these results, we also tested the activity of GA-OH in a panel of cervical cancer (CC) cell lines. These cells were good controls, since HPV is an established causative agent in CC carcinogenesis. Two HPV^+^ cell lines, SiHa and CaSki were selected as the positive controls and the Saos-2 cell line was used as an HPV-negative control. A similar pattern in selectivity differentiated by HPV status of the cell line was also observed (Figure 7B). For assessing the long-term effects on the survival of cells following GA-OH treatment, we performed the colony formation assay (CFA). Two cell lines, SCC19 (HPV^-^) and SCC104 (HPV^+^) were used for this study. The cells were treated for 24 hours with GA-OH, and then seeded for assessment of colony formation. Cell survival data from this study mirrored the impact of GA-OH on cell viability of HPV^+^ and HPV^-^ cell lines. The number of colonies in the HPV^+^ cell line was significantly and dose-dependently reduced at every dose of GA-OH tested compared to the SCC19 cell line. On the other hand, the number of colonies in SCC19 did not exhibit significant reduction relative to their control except at high concentrations of GA-OH (Figure 7C). This shows that GA-OH treatment has long-lasting effects on the viability and subsequent survival of HPV^+^ cells as compared to HPV^-^ cells.

### GA-OH stabilizes p53 levels and induces apoptosis in HPV^+^ cells

**Figure 8:**
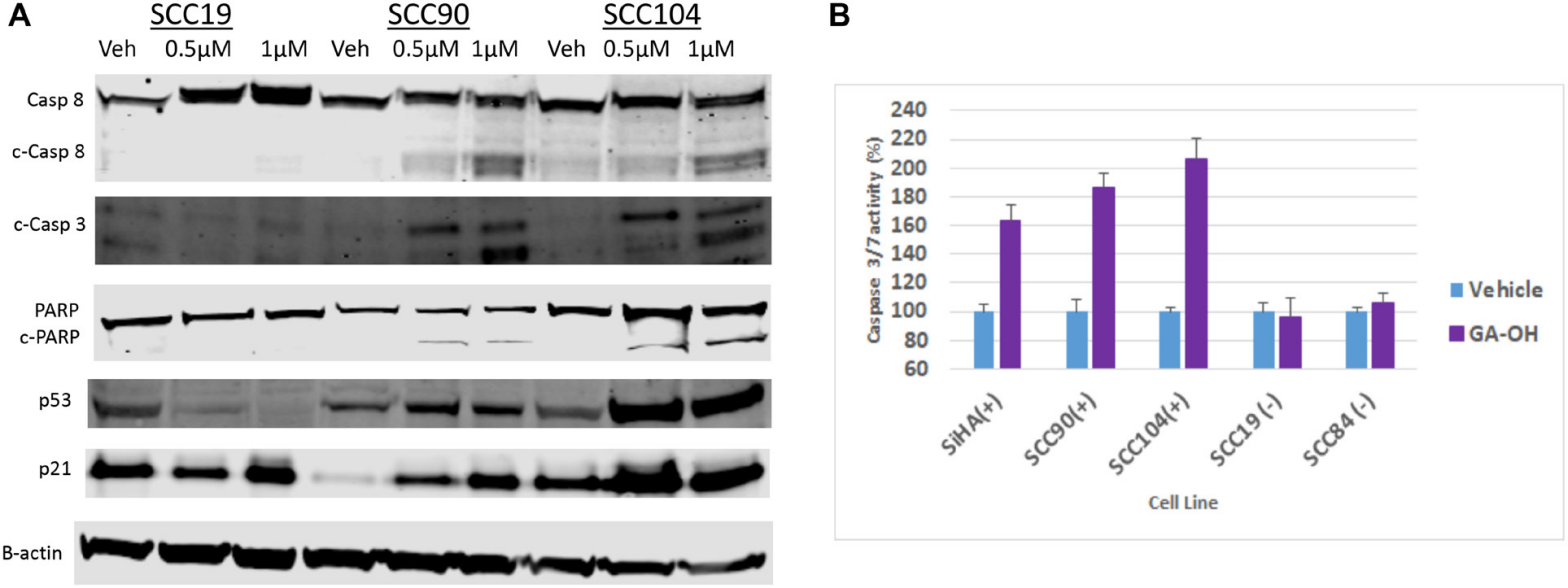
GA-OH activates p53 and Caspase 8 and induces apoptosis. (**A**) SCC19, SCC90 and SCC104 were seeded and treated with vehicle, 0.5 μM and 1 μM Of GA-OH for 24 hours. Activation of p53 was tested by blotting for p53 expression and activation. Caspase 8 activation and apoptosis was tested by blotting for Caspase 8 expression and activation of downstream targets. (**B**) Various HPV^+^ and HPV^-^ cell lines were seeded in 96 well plates and treated with 0.75 uM GA-OH. Caspase 3/7 activity was measured after 24 hours. HPV^+^ cell lines show high levels of Caspase 3/7 activity.

Based on the cell viability studies described above, as well as AlphaScreen™ data showing that that GA-OH prevents E6 from binding to both caspase 8 and the p53-recruiter, E6AP, we wondered whether activation of p53 and associated apoptotic effects could be contributing to the decrease in cell viability that we observed. We began by evaluating levels of p53 and its target gene product, p21, using immunoblotting. Treatment with GA-OH resulted in an increase of p53 in both the HPV^+^ SCC90 and SCC104 cell lines, but not their HPV^-^ counterpart (SCC19) (Figure 8A). These observations were corroborated by an induction of the levels of the target of p53, p21. The data shows a robust induction of p21 levels compared to the vehicle control. The basal levels of p53 in the HPV^+^ cell lines are lower compared to the HPV^-^ cell line, and this comes as no surprise given what is known regarding the effect of E6 on p53 stability [27].

We next looked at another target of E6, caspase 8, and the effect of GA-OH treatment on its activation and its downstream targets in the apoptosis cascade. Western blot analysis shows that GA-OH treatment leads to cleavage of caspase 8 in a dose-dependent manner. We noted an increase in caspase 8 levels in the HPV^-^ cell line upon exposure to GA-OH. However, there is no visible cleavage of caspase 8 itself (Figure 8A). A look at the down-stream effectors of apoptosis shows a similar trend. Caspase 3 is cleaved dose-dependently, as was observed with caspase 8. PARP is also cleaved, even though its cleavage is modest. Quantification of relative expression of each of the targets above using B-actin as a loading control is shown in Supplementary Figure 2.

We then confirmed the findings by conducting a Caspase 3/7 activity Glo assay, which is often utilized as a surrogate for apoptotic induction. Three HPV^+^ cell lines (SCC090, SCC104, SiHa) and two HPV^-^ cell lines (SCC19, SCC84) were assessed for activity of caspases 3 and 7, and thus for apoptosis activity. Significant apoptosis induction was observed in HPV^+^ cell lines compared to the controls (Figure 8B). Cell viability was also assessed in parallel to corroborate this result. Cells with higher apoptosis induction generally also showed higher reduction in cell viability as measured by MTT (Supplementary Figure 3). These results are consistent with the western blotting analysis involving caspase 8 and caspase 3, and also agree with the AlphaScreen™ data showing that GA-OH inhibits E6 binding to caspase 8. Also, as seen by immunoblotting, little to no apoptosis activity is observed in the caspase Glo experiment for the HPV^-^ cell lines. Collectively, these results indicate higher cell viability suppression by GA-OH in HPV^+^ versus HPV^-^ cell lines.

## DISCUSSION

In our previous published work, we identified a number of flavonoid compounds as E6-specific inhibitors by probing smaller chemical compound libraries [22, 24]. In this present study, our objective was to expand the chemical space in an effort to identify new and novel inhibitors of the HPV oncoprotein E6 that have greater potential for therapeutic development. We screened all compounds using our well-established AlphaScreen™ protocol, a protocol that has been successfully used as a HTS platform in drug discovery efforts for targeting protein-protein interactions [28, 29]. In the early stages of our screening workflow, a number of filtration steps and gates consistent with field standard practices were embedded to make the hit identification process appropriately rigorous. Our initial hit selection was based on criteria that a number of studies in the field have relied on, such as the statistically significant 3 Z-scores above the sample mean limit [30]. Moreover, hits that met this criterion were excluded if they did not exhibit at least ~50% inhibition of E6 binding, a cut-off that has also been employed in many studies. The primary hits we identified were then subjected to secondary assays for further filtration. In counter-screening, we chose hits with a selectivity index of at least 10; this minimum threshold is also generally regarded as a rigorous starting point for choosing compounds demonstrating specificity [31].

Allocation of the initial hit compounds into clusters based upon structural similarity revealed that the biggest cluster comprised flavonoid compounds. This finding was consistent with the literature, because most compounds that have been discovered thus far as E6 inhibitors have a flavonoid chemotype [32]. In fact, 8 of the compounds identified as initial hits in our study had already been published as E6 specific inhibitors. Gossypetin, baicalein and brazilin were found by Malecka K *et al.* to share a chromenon scaffold and to exhibit similar efficacy in blocking E6-mediated p53 degradation and cell growth in SiHa and HeLa cell lines [25]. Luteolin, another compound that was discovered through virtual screening and the filter-based *in vitro* assay by Baleja *et al.* as E6-directed inhibitor, was also found in our library as hit [26]. The other 4 molecules: myricetin, morin, kaempferol, quercetin had been discovered previously in our lab. Of these 4, myricetin had been validated as having the most activity in HPV-specific cell lines [22, 24]. Although we did not prioritize those compounds, due to known issues associated with drug development for flavonoid compounds, having such similar findings as previously published work has confirmatory value as it points to the robustness of our assay. Furthermore, these findings highlight the novelty and significance of our concurrent identification of GA and later, GA-OH, as molecules of interest.

It is important to note that two different sets of hands were involved in conducting this study. The primary screen and part of the counter-screening was done at Kansas University, while the second counter-screening and the rest of the experiments were done at Loma Linda University. There was repetition of experiments by both parties, and strong correlation of the results obtained, including parameters such as IC_50_ values from dose response experiments, attest to the fidelity of the experimental results. Finally, PAINS detection, visual analysis and SARs were done to complement the aforementioned steps and helped to select GA-OH from our set of hit candidates [33, 34].

The efficacy of GA-OH was further demonstrated in a biological context through cell-based assays. The results show that GA-OH suppressed cell proliferation and killed cells in an HPV-dependent manner, consistent with the role of E6 in cell growth and inhibition of apoptosis induction. In addition, activation of mediators of apoptosis, including p53 were also observed, particularly in the HPV^+^ cell models. Taken together, we believe that our process of hit selection and characterization was relatively comprehensive for this stage of the process. More work is needed to follow-up these initial findings. First, more experiments will be needed for an extensive SAR study. This will involve obtaining more analogs and testing them in order to draw better conclusions *regarding* which structural substituents are crucial or tolerated for the activity of gambogic acid. The other benefit of a more extensive SAR study is that we can potentially discover even more potent analogs. We also need to fully dissect the relative importance of p53 and caspase 8 in mediating the effects of E6 on cell growth and apoptosis, and the role GA-OH plays in disrupting these pathways. It is possible that other cellular targets, in addition to those we investigated, could be effectors of GA-OH. For instance, the parent compound of GA-OH has been shown to be active in other cancers [35], though no such activities have been reported for GA-OH and our findings are the first to demonstrate enhanced activity against HPV^+^ cells. Our observations were consistent with the earlier gambogic acid findings, in that gambogic acid showed some activity against HPV^-^ cell lines, even though HPV^+^ cell lines were significantly more sensitive. Importantly, we observed GA-OH to be more potent and selective for HPV^+^ versus HPV^-^ cells as compared to gambogic acid, suggesting that it has more specificity than its parent compound. We could not find significant information in the literature on the activity of GA-OH in other cell lines. However, GA-OH has improved solubility and we can speculate that the additional hydroxyl group it has most likely contributes to overall activity by providing another handle for hydrogen bonding between the molecule and E6. In addition, there is a high possibility that the extra polar group strengthens the hydrogen bond network that has been observed between small molecules and E6 [26, 36]. In terms of the safety profile, we *believe it unlikely* that the additional hydroxyl group will decrease the tolerability of GA-OH relative to GA. GA has been found to be relatively tolerable in animal studies, and toxicities to organs were only observed at high concentrations [37, 38]. Collectively, the evidence we have gathered suggests that GA-OH can serve as a basis for better understanding the mechanism of E6 inhibition, and has the potential for further development to improve potency and drug-likeness. Tools such as computational modelling and medicinal chemistry can utilize this inhibitor as a good starting point for optimization and in understanding important interactions between E6 and its inhibitors.

## MATERIALS AND METHODS

### Purification and preparation of proteins

Plasmids carrying E6 and caspase 8 (pGEX-E6 and pTriEx-Caspase 8) were previously constructed [22, 24]. Expression of GST-E6, GST-Caspase 8 and His_6_-Caspase 8 in *E. coli* and subsequent purification were carried out as previously described [22, 24]. GST-E6, GST-Caspase 8 and His_6_-Caspase 8 proteins were diluted into GST protein buffer (PBS pH 8.0, 5% glycerol, 2 mM DTT) and His protein buffer (20 mM HEPES pH 7.4, 150 mM NaCl, 2 mM KCl, 5% glycerol, 2 mM DTT), respectively. The concentration of the proteins was determined using Coomassie Plus – The Better Bradford Assay Reagent (Thermo Scientific, Waltham, MA, USA). Purity of the isolated proteins was assessed by sodium dodecyl sulfate-polyacrylamide gel electrophoresis (SDS-PAGE) separation and Coomassie staining.

### Compound library collection

The library we used for our screening comprised 3 sub-libraries for a total of about 5040 small molecule compounds. The Prestwick Chemical Library contains 1200 small molecules. Many compounds in this library possess drug-likeness properties (bioavailability and safety in humans), because 90% of the compounds are previously or currently marketed drugs, while 10% are bioactive alkaloids or related substances. The Microsource Spectrum Collection consisted of 2000 small molecules with a wide range of biological activities and structural diversity. Some of the compounds were known drugs, while others were natural products and non-drug enzyme inhibitors with pharmacological profiles not yet well characterized. The remainder were synthetic compounds that were uniquely synthesized by the Kansas University Chemistry Core as well as the Center for Chemical Methodology and Library Methodology.

### Primary library screening and initial hit selection

In total, our collection of compounds contained 5040 small molecules from 3 structurally diverse compound libraries (*see* Supplementary Table 1 for more details). The compounds were diluted to a working concentration in DMSO and screened at a single-point final concentration of 10 μM with no replicates. Briefly, 75 nL of each compound was transferred and added to wells of the destination 384-well plate using the Echo dispenser to 4 μL of blocking solution. 4 μL of 800 nM GST-E6 and 4 μL of His_6_-Caspase 8 were added and pre-incubated at room temperature for 60 minutes. 8 μL of the donor and acceptor bead mixture (final concentration of 20 μg/ml) was then added. The plates were sealed and incubated for 4 hours at room temperature before the plates were read using the EnvisionTM Multi-Label plate reader (Perkin Elmer Inc.). Percent inhibition for each compound was calculated, and the % inhibition value that was 3 standard deviations (SD) above the sample mean (μ+3SD) was used as the selection threshold. A 10-point serial dilution of these compounds was done for dose-dependency reconfirmation. Dose response inhibition curves were constructed and IC_50_ calculated using GraphPad Prism using four parameter non-linear regression analysis.

### Counter-screen assays and hit confirmation

The first counter-screen assay was based using the GST-His_6_ fusion peptide as the E6-binding partner instead of GST-E6-His_6_-Caspase 8. Hit candidate compounds from the primary screen were prepared using a 6-point serial dilution. Using an Echo dispenser, compounds were transferred to plates containing 4 μL of blocking buffer. 8 μL of 5 nM GST-His_6_ peptide substrate was then added. The mixture was pre-incubated at room temperature for 60 minutes. Glutathione donor and nickel chelate acceptor beads (final concentration 20 μg/mL) were added and incubated for another 60 minutes at room temperature. Dose-dependency of the compounds using GST-E6-His_6_-Caspase 8 was performed in parallel using the same protocol as in the primary screen. The signals were then read using the Envision™ plate reader. Following IC_50_ calculations using GraphPad Prism, selection was based on the Selectivity Index (SI) and a maximum inhibition of E6-caspase 8 binding ≥ 50%.

The second counter-screen was based on GST-Caspase 8 and His6-Caspase 8. Hits from the GST-6xHis counter-screen were tested in triplicate at a single concentration of 10 μM. Briefly, 5 μL of the compound was manually added to the plate wells containing 5 μL blocking buffer. 5 μL of 400 nM GST-Caspase 8 and 5 μL 400 nM His_6_-Caspase 8 were added and pre-incubated for 1 hr at room temperature. Glutathione donor and nickel chelate acceptor beads (final concentration 20 μg/mL) were added and incubated for another 60 minutes at room temperature before signal was quantified. This experiment was repeated 2 times on different days. Results were processed as described above, and % inhibition was calculated relative to the vehicle control. Compounds with % inhibition of caspase 8 dimerization less than 20% were chosen for further consideration.

### Cheminformatic filtering and cross-validation

Compounds that passed the two counter-screens were subjected to cheminformatic analysis as an additional filter for recognition and exclusion of compounds with problematic substructures. These substructures contain functional groups that may disrupt binding in many unrelated biochemical assays in a non-specific manner. Specifically, from the names and SMILES of the hit compounds we queried the following databases to find hits with pan assay interference (PAINS) patterns: Zinc15, SwissADME, FAFdrugs4 and PAINS-Remover. Compounds that made it onto the consensus list as having no PAINS patterns after filtering with these online tools were then selected. We then cross-validated the selected compound(s) with a related but different primary screen assay. Specifically, we replaced the caspase 8 used in the primary screen with E6AP and evaluated the inhibitory activity against E6 binding to E6AP (E6-E6AP) using the same steps as in the AlphaScreen™ protocol described above.

### Structure activity relationships (SARs)

Using the SciFinder and Zinc15 databases, we searched and identified several gambogic acid structural analogs. A subset of these analogues was commercially available and we purchased 8. The 8 analogs were obtained as follow: gambogenic amide (Enzo Life Sciences), gambogenic acid (Selleckchem), morellic acid (Aobious), 30-hydroxy gambogic acid (Quality Phytochemicals, LLC), acetyl gambogic acid (Microsource), and gambogin, neogambogic acid and isomorellinol (MolPort Natural Products). Additional gambogic acid was purchased from Tocris. The interactions of the analogues with E6-caspase 8 was tested using AlphaScreen™ technology using the same protocol as with the primary screen, and were compared to the parent compound. The effect on cell viability was also similarly done in HPV^+^ and HPV^-^ cell lines *via* the MTT assay (see below) and potency was determined using GraphPad IC_50_ curve fitting.

### Cell culture

Saos-2, SiHa, and CaSki cells were obtained from the America Type Culture Collection (Manassas, VA, USA). SiHa and CaSki were cultured in Eagle’s minimal essential medium (Invitrogen, Carlsbad, CA, USA) as described previously. HNSCC cell lines were obtained from several sources: UM-SCC47-TC-Clone 3 (#47CL3), UPCI-SCC90-UP-Clone 35 (#90), and SCC 84 were a gift from Dr. John Lee, Sanford Research (South Dakota, USA). UMSCC 19 (#19), UMSCC 29 (#29), UMSCC49 (#49) and UMSCC 104 (#104) were a gift from Dr. Thomas Carey, University of Michigan (Michigan, USA). UPC1-SCC152 was purchased from ATCC. HNSCC cells were cultured in Dulbecco’s Modified Eagle Medium (Mediatech, Manassas, VA, USA) supplemented with 10% of FBS. Saos-2 cells were grown in McCoy 5a medium, and HCT116 cells were cultured in RPMI medium supplemented with 10% FBS.

### MTT cell viability assays

All working concentrations were diluted in PBS to the desired concentration before use. To test the effect of gambogic acid and/or its derivatives on cell viability, all cell lines were seeded at 2 × 10^4^ per well in 96-well plates and allowed to adhere overnight. Various concentrations of the analogues were added and the cells incubated at 37°C for 24 hr. Viability was then measured using the MTT assay, performed as described previously [22]. All experiments were repeated at least three times (three biological replicates, carried out on different days). Data presented are from a representative experiment. Cell viability and potency were assessed from % inhibition relative to the vehicle control, and IC_50_ dose curves were generated using GraphPad Prism.

### Caspase activity assay

Cells were seeded into white walled 96-well plates at 2 × 10^4^ cells per well in 100 μL media and incubated overnight. GA-OH (0.75 μM) and vehicle were then added and incubated at 37°C for 24 hr. Caspase 3/7 activity was measured using the Caspase 3/7 Glo kit (Promega, Fitchburg, WI, USA) following the manufacturer's instructions. Briefly, room temperature-equilibrated Caspase-Glo reagent was added (Promega) to each well. The plate was mixed by placing it on an orbital shaker and incubated for 30 secs at room temperature, then incubated at room temperature. After a 2-hr incubation, luminescence was measured using a plate-reading fluorimeter (Flx800, Bio-Tek Instrument Co., Winooski, VT, USA). Background activity (blank reaction) was subtracted from all experimental wells. Percent activity of caspase 3/7 in wells treated with GA was then expressed relative to vehicle treated wells.

### Western blotting

Adherent cells were washed with ice cold PBS. Cell lysis buffer containing protease inhibitor cocktail was added and cells were scraped off into a tube on ice. The cells were incubated on ice for 10 minutes. Cell lysates were separated by SDS-PAGE and electrophoretically transferred to PVDF membranes. Following blocking, antibodies directed against caspase 8, p53, cleaved PARP, cleaved caspase 3, p21, and β-actin (Cell signaling) were applied at 1:5000 dilution. Anti-mouse and anti-rabbit secondary antibodies were then employed (LI-COR Biosciences, Lincoln, NE, USA). Signals were measured using the Odyssey Infrared Imaging system (LI-COR Biosciences) and quantified using Image J.

### Colony formation assay

Sub-confluent monolayer cells were treated with different doses of GA-OH for 24 hours. Cells were trypsinized and re-suspended before re-plating into 6 well plates in DMEM or MEM at 500–1000 cell densities, depending on the cell line. Cells were then allowed to grow for 10–20 days, depending on the cell line, before fixing and staining. A mixture of methanol/acetic acid was used for fixing, followed by 0.5% crystal violet staining. Plates were imaged using UV imager, and colonies with more than 50 colonies counted using image J. Surviving fractions were determined by dividing the number of colonies by the number of cells seeded as a product of the corresponding plating efficiency. Survival fractions curves were plotted using GraphPad Prism.

### Data analysis

Binding and dose-response curves were fitted using GraphPad software (GraphPad Software, Inc., La Jolla, CA, USA).

Z’ factor was calculated from intraplate controls as previously described using the formula [30]:

*Z’* = 1–(3 × STDEV_Control_ + 3 × STDEV_Background_)/(Mean_Control_–Mean_Background_) where STDEV is the standard deviation and control is 0% inhibition (maximum signal) and background is 100% inhibition (minimum signal).

Signal to background ratio was determined as follows:

*S/B ratio* = Mean_control_/Mean_background_

Percent (%) activity and Percent (%) inhibition of binding for the compounds was calculated from Alpha Screen signals using the equations:

*Percent (%) activity*: 100 (Mean_compound_–Mean_background_)/(Mean_control_–Mean_background_)

*Percent (%) inhibition*: 100–% activity.

*Percent Coefficient of Variation:* (CV%) = 100 × (STDEV/Mean)

*Selectivity Index* (SI): IC50_GST-His peptide_/IC50_E6-Caspase8_ *≥* 10

*Hit Selection Threshold: ≥* μ + 3SD where μ is the sample mean and SD is standard deviation.

## SUPPLEMENTARY MATERIALS



## Abbreviations

GA: gambogic acid
GA-OH: 30-hydroxygambogic acid
HNSCC: Head and neck squamous cell carcinoma, HPV: Human papillomavirus
PAINS: pan assay interference
PPI: protein-protein interaction
SI: Selectivity Index
SAR: Structure activity relationship
