# Supplementary material for: A high-content AlphaScreen™ identifies E6-specific small molecule inhibitors as potential therapeutics for HPV+ head and neck squamous cell carcinomas
Source: Oncotarget. 2021 Mar 16;12(6):549–61. doi: 10.18632/oncotarget.27908 (PMC7984827; doi:10.18632/oncotarget.27908)
Supplement: Supplementary file 1 [file oncotarget-12-549-s001.pdf]

A high-content AlphaScreen™ identifies E6-specific small molecule inhibitors as potential therapeutics for HPV$^+$ head and neck squamous cell carcinomas

SUPPLEMENTARY MATERIALS

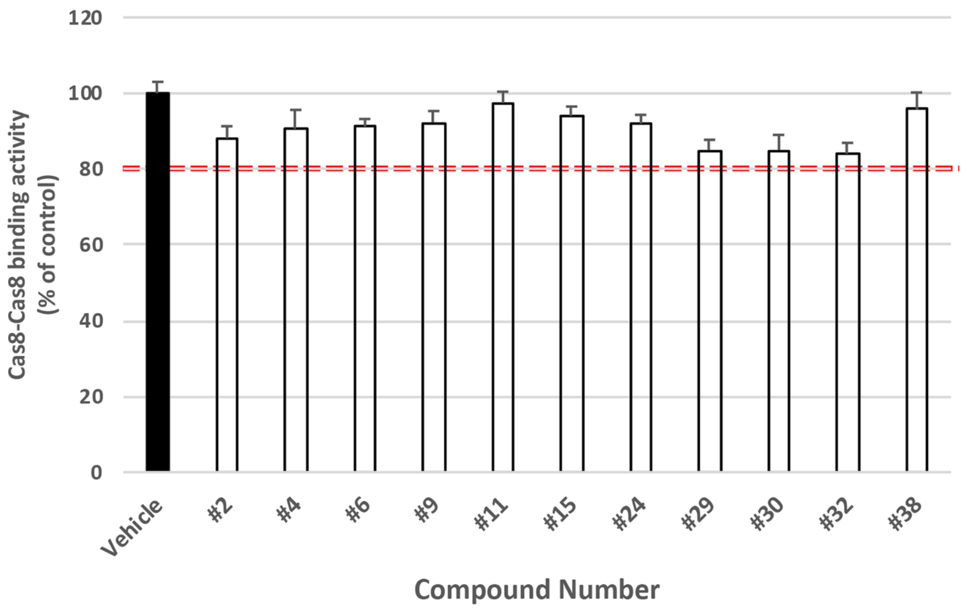

Supplementary Figure 1: Second counter-screening of the initial hits. The 11 compounds that exhibited less than 20% inhibition of Caspase 8-Caspase 8 binding (at 10 µM) as shown above were selected as true hits for further analysis. The dotted red line indicates the pre-defined threshold for selection for downstream analysis.

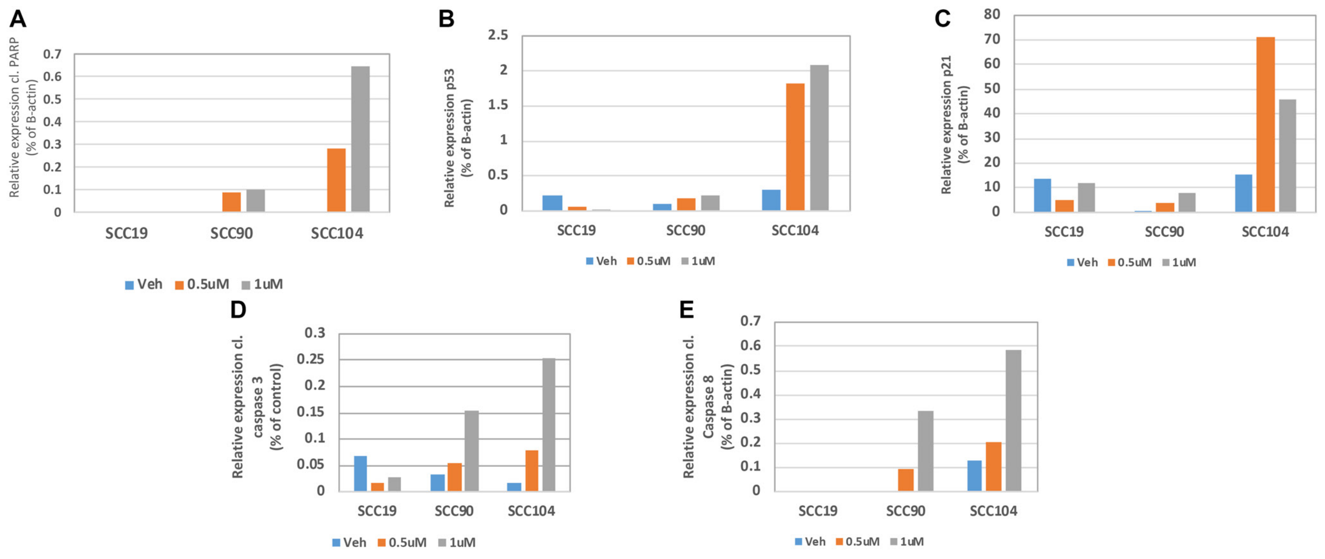

Supplementary Figure 2: Quantification of expression of protein levels of cleaved PARP (**A**), p53 (**B**), p21 (**C**), cleaved caspase 8 (**D**), and cleaved caspase 3 (**E**) relative to B-actin expression.

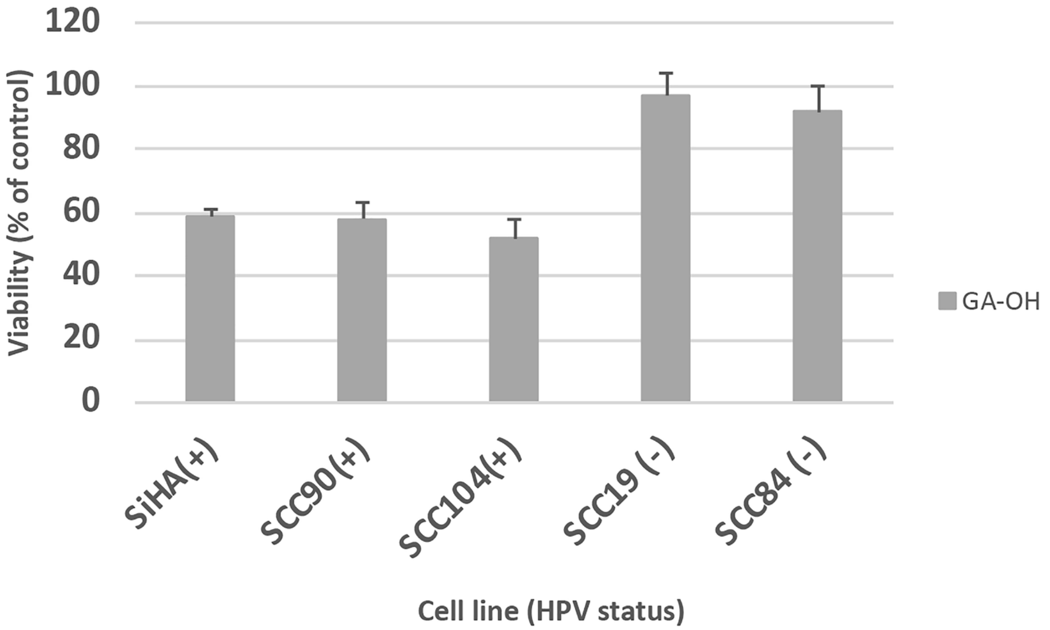

Supplementary Figure 3: Cell % viability as measured by MTT. Cells used in the Glo assay were treated with GA-OH in a corresponding 96-well plate, and viability was assessed by MTT after 24 hrs.

Supplementary Table 1: Content of the 3 libraries used in the screen

Library	No. of compounds	Tanimoto Index	Type of compounds	Description
CMLD	1840	0.902	Diversity, drug-like	Diverse 3D pharmacophores used for selection. Over 60 proprietary chemical filters applied (including Lipinski's rule of 5) and Daylight Tanimoto similarity measures to ensure structural diversity and drug-likeness.
Prestwick	1200	0.81	FDA Approved drugs	Marketed drugs
Microsource spectrum	2000	0.80	Bioactives (560 compounds), FDA approved (640 drugs), Natural products (800 compounds)	75% of the natural product collection encompasses: alkaloids (16%), flavanoids (12%), sterols/triterpenes (12%), diterpenes/sesquiterpenes (10%), benzophenones/chalcones/stilbenes (10%), limonoids/quassinoids (9%), and chromones/coumarins (6%).The remainder 25% of the collection includes includes quinones/quinonemethides, benzofurans/benzopyrans, rotenoids/ xanthones, carbohydrates, and benztropolones/depsides/depsidones, in descending order.

Supplementary Table 2: IC_{50} (µM) values against E6-Caspase 8 binding for the 96 compounds that were selected as initial hits following the primary screen

Compound ID	IC_{50} (µM)	Compound ID	IC_{50} (µM)	Compound ID	IC_{50} (µM)	Compound ID	IC_{50} (µM)
KU0101350	0.08	KU0101639	1.71	KU0102344	4.85	KU0103230	2738.00
KU0101521	0.30	KU0103046	1.81	KU0102544	4.92	KU0105079	1.87E+28
KU0101407	0.35	KU0101640	2.07	KU0102272	4.99	KU0103392	~ 2.215e+008
KU0101633	0.57	KU0101863	2.09	KU0102576	5.15	KU0105025	~ 2.518e+007
KU0101351	0.57	KU0101505	2.26	KU0102217	5.21	KU0103558	~ 21210
KU0101562	0.64	KU0102051	2.32	KU0102474	5.79	KU0104995	~ 28789
KU0101152	0.76	KU0101513	2.45	KU0102410	6.08	KU0103640	~ 3.507e+006
KU0101570	0.76	KU0101869	2.45	KU0102635	6.12	KU0104819	~ 35931
KU0101353	0.88	KU0101837	2.46	KU0102227	6.18	KU0103575	~ 378185
KU0101587	0.98	KU0102558	2.75	KU0102735	7.13	KU0105084	~ 4.523e+008
KU0101202	1.15	KU0101845	2.76	KU0102371	7.14	KU0103589	~ 4.965e+013
KU0101667	1.23	KU0102486	2.78	KU0102799	7.17	KU0104990	~ 6.513e+007
KU0101354	1.29	KU0101853	2.89	KU0102165	7.30	KU0103518	~ 8170
KU0101484	1.33	KU0102566	3.03	KU0102807	7.76	KU0105263	Ambiguous
KU0101228	1.35	KU0101965	3.27	KU0102510	8.90	KU0103598	Ambiguous
KU0101870	1.37	KU0103086	3.48	KU0102848	9.08	KU0106020	Ambiguous
KU0101357	1.43	KU0102126	3.58	KU0044882	9.15	KU0104019	Ambiguous
KU0101886	1.47	KU0103094	3.59	KU0104071	10.74	KU0106101	Ambiguous
KU0101558	1.49	KU0102278	3.92	KU0102753	10.74	KU0103872	Ambiguous
KU0101902	1.592	KU0102431	4.189	KU0103920	10.9	KU0106021	Ambiguous
KU0101566	1.618	KU0102151	4.287	KU0102777	10.92	KU0103810	Ambiguous
KU0101958	1.619	KU0102551	4.358	KU0104003	37.07	KU0106102	Ambiguous
KU0101598	1.62	KU0103055	4.701	KU0103302	37.55	KU0104231	Ambiguous
KU0101982	1.673	KU0102512	4.843	KU0104381	58.77	KU0106103	Ambiguous

The 69 hits selected for counter-screening are highlighted in gray.

Supplementary Table 3: IC_{50} (µM) values against E6-Caspase 8 binding for the 11 compounds that passed counter-screening

Hit Compound No.	IC_{50} (µM)
#2	3.59
#4	1.48
#6	6.06
#9	2.94
#11	1.80
#15	1.39
#24	2.00
#29	0.61
#30	6.23
#32	1.46
#38	5.17
